# Molecular Mechanisms of Ferroptosis and Relevance to Cardiovascular Disease

**DOI:** 10.3390/cells11172726

**Published:** 2022-09-01

**Authors:** Lai-Hua Xie, Nadezhda Fefelova, Sri Harika Pamarthi, Judith K. Gwathmey

**Affiliations:** Department of Cell Biology and Molecular Medicine, New Jersey Medical School, Rutgers University, Newark, NJ 07103, USA

**Keywords:** ferroptosis, cardiovascular disease, iron, ROS, lipid peroxidation, mitochondria

## Abstract

Ferroptosis has recently been demonstrated to be a novel regulated non-apoptotic cell death characterized by iron-dependence and the accumulation of lipid peroxidation that results in membrane damage. Excessive iron induces ferroptosis by promoting the generation of both soluble and lipid ROS via an iron-dependent Fenton reaction and lipoxygenase (LOX) enzyme activity. Cytosolic glutathione peroxidase 4 (cGPX4) pairing with ferroptosis suppressor protein 1 (FSP1) and mitochondrial glutathione peroxidase 4 (mGPX4) pairing with dihydroorotate dehydrogenase (DHODH) serve as two separate defense systems to detoxify lipid peroxidation in the cytoplasmic as well as the mitochondrial membrane, thereby defending against ferroptosis in cells under normal conditions. However, disruption of these defense systems may cause ferroptosis. Emerging evidence has revealed that ferroptosis plays an essential role in the development of diverse cardiovascular diseases (CVDs), such as hemochromatosis-associated cardiomyopathy, doxorubicin-induced cardiotoxicity, ischemia/reperfusion (I/R) injury, heart failure (HF), atherosclerosis, and COVID-19–related arrhythmias. Iron chelators, antioxidants, ferroptosis inhibitors, and genetic manipulations may alleviate the aforementioned CVDs by blocking ferroptosis pathways. In conclusion, ferroptosis plays a critical role in the pathogenesis of various CVDs and suppression of cardiac ferroptosis is expected to become a potential therapeutic option. Here, we provide a comprehensive review on the molecular mechanisms involved in ferroptosis and its implications in cardiovascular disease.

## 1. Introduction

**Figure 1:**
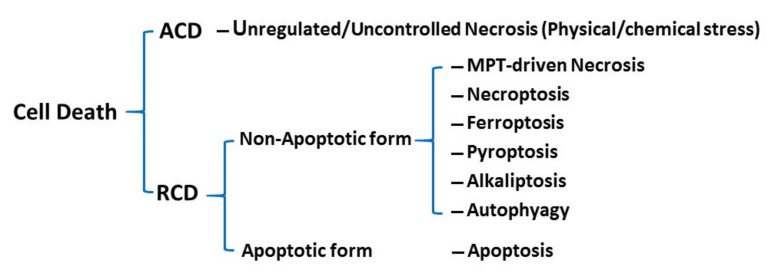
Classification of cell death. ACD: accidental cell death; RCD: regulated cell death; MPT: mitochondrial permeability transition pore.

Although cell death is an often necessary process in multicellular organisms during development and can serve as a defense against the development of cancer and other hyperplasic diseases, it can also play a pivotal role in causing disease in many organs including the heart [1,2]. Several forms of cell death have been discovered and have been divided into two categories, i.e., accidental cell death (ACD) and regulated cell death (RCD) (Figure 1). ACD is a passive process in which uncontrolled necrosis is the major type. In contrast, RCD is an active process that plays an important role in both regulation of tissue homeostasis and in the pathogenesis of various diseases. There are many types of RCD, including apoptotic and non-apoptotic forms. A growing number of new non-apoptotic RCD forms such as necroptosis, ferroptosis, pyroptosis, and autophagy-dependent cell death have recently been identified [3]. The forms of RCD have different morphological, molecular, biochemical, and functional features [3,4]. Ferroptosis is a recently revealed novel regulated non-apoptotic cell death characterized by its iron-dependence and the accumulation of lipid peroxidation, which causes membrane damage. According to the latest recommendations by the Cell Death Nomenclature Committee in 2018 [3], ferroptosis is “a form of regulated cell death initiated by oxidative perturbations of the intracellular microenvironment that is under constitutive control by glutathione peroxidase 4 (GPX4) and can be inhibited by iron chelators and lipophilic antioxidants”. In this review, we attempt to: (1) update current literature on the proposed molecular mechanisms involved in ferroptosis; and (2) to point out the possible clinical relevance of ferroptosis in cardiovascular disease. The reader may also refer to recent comprehensive review articles on ferroptosis [5,6].

## 2. Hallmarks of Ferroptosis

### 2.1. Biochemical Properties

The concept of “ferroptosis” was first proposed by Dixon in 2012 [7]. Ferroptosis is an iron-dependent and lipid peroxidation-driven cell death process. Therefore, both iron accumulation and increased lipid reactive oxygen species (ROS) play essential roles in regulating the process of ferroptosis [7,8,9]. Iron is an important trace element that participates in numerous biological processes under physiological conditions [10,11]. However, it can also play a central role in oxidative stress being capable of becoming toxic. As there is no specific physiologic means to excrete iron, iron accumulation is important in that it can lead to multi-systemic disease and organ dysfunction. How is iron accumulation linked to ferroptosis? First, iron promotes excessive production of ROS via the Fenton reaction and can increase intracellular as well as lipid oxidative stress levels [7,12,13,14,15]. Second, iron participates in the activation of lipoxygenases (LOX), which are iron-containing enzymes that promote the generation of lipid ROS (e.g., lipid peroxides) [16,17]. Increased lipid peroxidation subsequently leads to cellular as well as mitochondrial membrane damage and rupture resulting in ferroptosis [18].

The most important hallmark of ferroptosis is lipid peroxidation, which is a free radical-driven, iron-dependent (Fenton reaction) and LOX-catalyzed degradation of lipids (especially polyunsaturated fatty acids (PUFAs)) and cholesterol in cytoplasmic or organelle membranes [19,20,21]. The production of malondialdehyde (MDA) and 4-hydroxynonenal (4-HNE) (two major by-products of lipid peroxidation) are found to be increased during ferroptosis and can be measured as indicators of lipid peroxidation [22]. A newly-developed specific lipid peroxide probe Liperfluo (Dojindo Molecular Technologies, Rockville, MD, USA) has been used to measure lipid peroxidation [21,23,24].

On the other hand, lipid peroxides can be reduced to lipid alcohol (i.e., L-OH) by glutathione peroxidase 4 (GPX4), which is a key regulator of lipid peroxidation using glutathione (GSH) as a reducing substrate. Reduction in the availability and activity of GPX4 results in the accumulation of membrane lipid peroxides, oxidative lipid damage, and subsequently ferroptosis [7,25,26].

The overexpression of some genes/proteins that are involved in lipid metabolism have been used as biomarkers of ferroptosis. These include (1) prostaglandinendoperoxide synthase 2 (PTGS2/COX2), a key enzyme in prostaglandin biosynthesis [21]; and (2) Acyl-CoA synthetase long-chain family member 4 (ACSL4), an enzyme that enhances the PUFA content in membrane phospholipids [19,20].

### 2.2. Morphological Properties

Many studies have shown that cells undergoing ferroptosis exhibit necrosis-like morphological changes, such as a loss of plasma membrane integrity, cytoplasmic swelling, cell membrane rupture, and the release of intracellular content [2,27,28,29]. This is different from the condensation of nuclear chromatin and the formation of apoptotic bodies in the cytoplasm that is seen with apoptosis [30] or the formation of cytoplasmic double membrane vesicles noted in autophagy [31]. Furthermore, ferroptotic cells often show significant changes in mitochondrial morphology, such as shrunken (smaller) mitochondria, increased mitochondrial membrane density, reduced or absent crista, and rupturing of the mitochondrial outer membrane [7,26,32].

## 3. Molecular Mechanisms/Pathways of Ferroptosis

**Figure 2:**
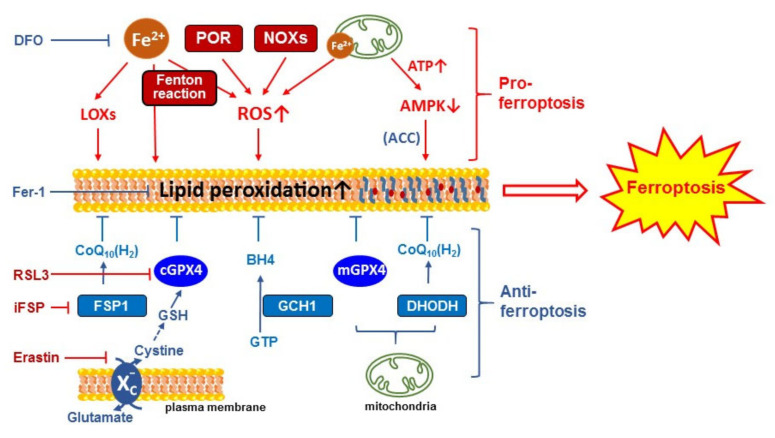
Regulatory mechanism of ferroptosis. Ferroptosis is induced by iron-dependent lipid peroxidation in plasma or mitochondrial membrane. Pro-ferroptosis (red) and anti-ferroptosis (blue) pathways are shown. Well-used ferroptosis inducers (e.g., RSL3, iFSP, and Erastin) and inhibitors (e.g., DFO and Fer-1) and their targets are indicated. The accumulation of cytosolic and mitochondrial iron, increased ROS, and lipid peroxidation promote ferroptotic cell death. DFO: deferoxamine; POR: P450 oxidoreductase; NOX: NADPH oxidases; LOX: lipoxygenase; ROS: reactive oxygen species; AMPK: AMP-activated protein kinase; ACC: acetyl-CoA carboxylase; Fer-1: ferrostatin-1; CoQ10: coenzyme Q10; RSL3: RAS-selective lethal 3; GPX4: glutathione peroxidase 4; FSP1: ferroptosis suppressor protein 1; DHODH: dihydroorotate dehydrogenase; GSH: glutathione; GCH1: GTP cyclohydrolase 1; BH4: tetrahydrobiopterin.

Different forms of RCD require special molecular machinery to trigger cell death [2,4]. Understanding pathways involved in ferroptosis have historically been approached from a perspective of leveraging them for killing cancer cells [25,33]. The induction of ferroptosis may be used as a promising anticancer strategy [7,8]. However, ferroptosis turns to a foe with regard to neurodegenerative and heart diseases [5,6]. Despite recent intensive studies by oncology and neuroscience groups, the study of ferroptosis in the heart is only just emerging [34,35,36,37,38,39]. Here, we review the major pathways (Figure 2) that have been discovered which can result in ferroptosis in different cell types including cancer, neuronal, and cardiac cells. It should be noted that differences in ferroptotic and anti-ferroptotic pathways and key regulatory pathways may exist between different cell types or organs.

### 3.1. System x_c_^−^-GSH-GPX4 Pathway

The phenomenon of ferroptosis was originally found while screening small molecular compounds for cancer treatment [40,41]. Erastin and RAS-selective lethal 3 (RSL3) were identified to selectively kill RAS-mutant cancer cells depending on their ability to induce a new type of iron and lipid peroxidation-dependent cell death [7]. Now, it is known that erastin is a blocker of the cystine/glutamate transporter (system x_c_^−^, gene: SLC7A11), while RSL3 is an inhibitor of GPX4, both of which play important roles in the lipid peroxidation process and can induce ferroptosis.

System x_c_^−^ is an amino acid transporter expressed in the plasma membrane that mediates the uptake of cystine in exchange for intracellular glutamate. Intracellular cystine is then reduced to cysteine, which is a rate-limiting precursor in glutathione (GSH) synthesis. GSH is then used as a cofactor for GPX4, which acts as a lipid hydroperoxidase to reduce lipid peroxide (i.e., L-OOH) to lipid alcohol (i.e., L-OH) [42,43]. Therefore, GPX4 is one of the important defense mechanisms by which cells detoxify lipid peroxides. Normal GPX4 activity is essential to maintain membrane lipid homeostasis, preventing excess accumulation of toxic lipid peroxide and free radical formation (i.e., L-OOH and L-O^•^), thereby attenuating ferroptosis [7,44]. Reduced GSH also has an antioxidant effect.

There are three isoforms of GPX4, i.e., mitochondrial (mGPX4), cytosolic (cGPX4), and nuclear GPX4 (nGPX4) [45,46,47]. While it has been well accepted that cGPX4 plays an essential role in reducing plasma membrane lipid peroxide and attenuating ferroptosis, the role of mGPX4 in reducing mitochondrial membrane lipid peroxidation and defending against ferroptosis has been in debate [35,48,49]. Recent progress regarding mitochondrial regulation of ferroptosis is discussed in Section 3.4. Taken together, the inhibition of system x_c_^−^ (e.g., by erastin), a decreased GSH level, and inactivation of GPX4 by either pharmacological (e.g., by RSL3) or genetic approaches all lead to the accumulation of lipid ROS and the occurrence of ferroptosis [7,25,26].

### 3.2. Iron, Fenton Reaction and Formation of Intracellular and Lipid ROS

Iron is an essential biological element for normal cellular homeostasis and plays an important role in a wide range of physiological processes including red blood cell production, oxygen transport, electron transport, DNA synthesis, and lipid metabolism [50]. However, iron can become toxic if levels become too high, i.e., under iron overload conditions where it may cause dysfunction of multiple organs, including the heart. Readers may refer to our and other’s recent review articles for details on iron metabolism and iron overload-related cardiomyopathy [12,51]. Iron overload-induced cardiomyocyte death has been attributed to ferroptosis [34,52,53]. During ferroptosis, lipid peroxidation occurs in an iron-dependent manner. Although the exact mechanisms for the iron dependence is controversial, the activation of LOX and production of ROS (including lipid radical formation) via the Fenton reaction may offer some explanations.

#### 3.2.1. Cellular ROS Production

**Figure 3:**
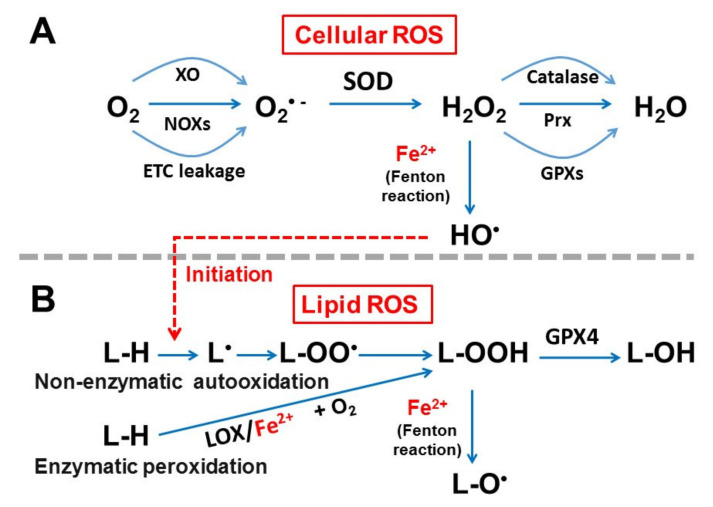
Generation of cellular (**A**) and lipid (**B**) ROS. See text for details. XO: xanthine oxidase; NOXs: NADPH oxidases; ETC: electron transport chain; O_2_^•−^: superoxide; SOD: superoxide dismutase; H_2_O_2_: hydrogen peroxide; Prx: peroxiredoxin; GPXs: glutathione peroxidases; HO^•^: hydroxyl radical; L-H: lipid; L^•^: lipid radical; L-OO^•^: lipid peroxyl radical; L-OOH: lipid peroxides; L-O^•^: lipid alkoxyl radicals; L-OH: lipid alcohol; LOX: lipoxygenase.

Cellular (soluble) ROS including superoxide (O_2_^•−^), hydrogen peroxide (H_2_O_2_), and hydroxyl radicals (HO^•^) are highly reactive molecules that are thought to exert both physiological and pathophysiological effects [54] (Figure 3A). The main source of soluble ROS in cardiomyocytes comes from O_2_^•−^ whose formation is mainly through the mitochondrial electron transport chain (ETC), NADPH oxidases (NOXs), and xanthine oxidase (XO). Under normal conditions, ROS is kept at physiological levels by a number of antioxidative enzymes. For example, superoxide dismutase (SOD) catalyzes the degradation of O_2_^•−^ to form less reactive H_2_O_2_ and O_2_. The resulting H_2_O_2_ is in turn reduced to H_2_O and O_2_ by other complementary enzymatic scavenging systems such as catalase, peroxiredoxin (Prx), and glutathione peroxidases (GPXs). When the balance between the production of oxidants and antioxidants is disturbed, intracellular ROS levels can be elevated, leading to oxidative stress, which in turn exerts numerous pathological effects thereby damaging DNA, proteins, and lipids [25,55,56]. ROS has been implicated in the pathogenesis of ischemia-reperfusion injury, heart failure (HF), hypertension, cardiac hypertrophy, and arrhythmias [55,56,57,58].

#### 3.2.2. Fenton Reaction and Generation of Cellular and Lipid ROS

One of the most damaging effects of iron is catalyzing Fenton reactions (using Fe^2+^) to generate highly toxic free radicals, i.e., hydroxyl radicals (HO^•^) from H_2_O_2_ [13,14,15]. It has also been shown that the overall ROS levels are higher under iron overload conditions [59]. We have also provided evidence that both cytosolic (measured with DCF) and mitochondrial (measured with MitoSOX Red) ROS levels are increased after acute or chronic iron treatment [12,53].

Lipid ROS include lipid peroxyl radical (L-OO^•^), lipid peroxides (L-OOH), and lipid alkoxyl radicals (L-O^•^), which are formed through either non-enzymatic autoxidation or enzymatic lipid peroxidation pathways (Figure 3B) [43,60]. There are three steps in the non-enzymatic autoxidation of certain lipids (e.g., PUFA and cholesterol), i.e., initiation, propagation, and termination [43]. The initiation step is dependent on intracellular ROS, e.g., soluble HO^•^ that abstracts a hydrogen atom from lipids to generate a lipid radical (L^•^). In the propagation step, the lipid radical reacts with oxygen (O_2_) to form a peroxyl radical L-OO^•^, which can then abstract hydrogen atoms from other lipids to form lipid peroxides (L-OOH). Importantly, highly reactive alkoxyl radicals L-O^•^ can be generated from L-OOH also via the Fenton reaction, which is reduced iron (Fe^2+^)-dependent. In the termination step, radical species spontaneously react to form non-radical dimers.

On the other hand, the enzymatic lipid peroxidation process is catalyzed by lipoxygenase (LOX), which is an iron-containing enzyme and can also participate in the formation of certain types of lipid peroxides (L-OOH) [21,60,61]. It has been suggested that arachidonic acid and linoleic acid are the most abundant polyenoic fatty acids that serve as substrates for LOX [21]. It should be noted that iron is required for the activity of LOX enzymes that use oxygen to form hydroperoxyl groups at different carbon positions of acyl chains. Some studies have shown that the inhibition of LOXs prevents ferroptosis in some models [17,62]; however, the universal link between LOX and ferroptosis is still under debate [43].

The iron-dependent nature of ferroptosis may, taken together, at least partly be explained by: (1) the generation of both soluble and lipid ROS involving iron-mediated Fenton reactions; and (2) lipid peroxidation driven by LOX (also POR, see below), which is also iron-dependent. Therefore, iron chelators (e.g., deferoxamine) might suppress the incidence of ferroptosis under various conditions [7,34,53].

### 3.3. Other Oxidative and Antioxidant Systems

It is notable that multiple oxidative and antioxidant systems are involved in lipid peroxidation during ferroptosis [63].

#### 3.3.1. POR and CYB5R1

As demonstrated in the original study by Dixon et al. and more recent publications, the NOX family enzymes make significant contributions to the generation of soluble and lipid ROS and the induction of ferroptosis in many cell types, including the heart [7,64]. In addition, recent studies have found that NADPH-cytochrome P450 oxidoreductase (POR) and NADH-cytochrome b5 reductase (CYB5R1) contribute to the initiation of ferroptosis in multiple cell lines [18,65]. Genetic deficiency of POR/CYB5R1 has been shown to block both lipid peroxidation and cell death [18,65]. POR is an endoplasmic-reticulum-residing oxidoreductase, which promotes the generation of H_2_O_2_. As discussed above, H_2_O_2_-derived hydroxyl radicals (HO^•^) (via the Fenton reaction) promote the initiation of lipid peroxidation and subsequently, the induction of ferroptosis.

#### 3.3.2. FSP1-CoQ10 Pathway

As mentioned above, endogenous antioxidant defense systems, such as the aforementioned system, the x_c_^−^ -GSH-GPX4 pathway, constantly combat oxidative stress and suppress lipid peroxidation and ferroptosis. Additionally, recent studies have discovered a parallel anti-oxidant protection system, i.e., the ferroptosis suppressor protein 1 (FSP1)-NADH-dependent coenzyme Q10 (CoQ10) pathway [66,67,68]. FSP1 functions as a CoQ10 oxidoreductase, which reduces CoQ10 (also known as ubiquinone) to CoQH_2_ (ubiquinol). CoQ10 is critical for electron transfer in the mitochondrial membrane for respiratory chain activity. In addition, when reduced, it forms CoQH_2_, which also acts as a lipophilic free-radical-trapping antioxidant (RTA) in the plasma membrane [69], thereby suppressing lipid peroxidation and ferroptosis. It has also been suggested that FSP1 can be used as a biomarker of resistance to ferroptosis. A FSP1 inhibitor (iFSP) can be used to induce ferroptosis or to sensitize cells to other ferroptosis inducers [68]. The recently discovered mitochondrial dihydroorotate dehydrogenase (DHODH)-CoQ10 defense system [70] (a counterpart of cytosolic FSP1-CoQ10 system) is discussed in Section 3.4.2 below.

In addition, recent studies have demonstrated that GTP cyclohydrolase 1 (GCH1) also suppresses ferroptosis via generation of tetrahydrobiopterin (BH_4_), another RTA [71,72]. It still needs to be validated whether and how this pathway is involved in cell death, especially with regard to ferroptosis in the heart and how it might be relevant to cardiovascular disease.

#### 3.3.3. Transcriptional Regulation

It has been well known that antioxidant defense processes are further regulated by nuclear transcriptional factors [73,74,75]. In particular, the activation of nuclear factor erythroid 2-like 2 (Nrf2) plays a major role in the suppression of ferroptosis [76,77]. Nrf2 is a stress-inducible transcription factor whose target genes (upregulation) include antioxidants (e.g., catalase, SOD, Prx), iron metabolism (e.g., Fth-1), and GSH synthesis. Therefore, Nrf2 is an important transcriptional regulator of anti-ferroptotic genes that are associated with the accumulation of free iron and lipid peroxidation. It has been shown that the knockdown of Nrf2 may sensitize cancer cells to ferroptosis [76,77].

### 3.4. Potential Roles of Mitochondria in Ferroptosis Regulation

Mitochondria are organelles that are referred to as energy factories of the cell since they conduct oxidative phosphorylation and generate most of the energy in the form of ATP in eukaryotic cells [78]. Meanwhile, as aforementioned, mitochondria are also the major source of ROS production. They also host many important metabolism processes such as the tricarboxylic acid (TCA) cycle and glutaminolysis.

It has been well known that mitochondria play pivotal roles in governing multiple forms of RCD [79]. However, previous studies on the role of mitochondria in ferroptosis have generated complex and somewhat controversial results. For example, some studies have suggested that there is no involvement of mGPX4 or mitochondrial lipid peroxidation in the induction of ferroptosis [26], while others have provided evidence linking mitochondria to ferroptosis [35,80]. Based on some recent findings [70,81,82,83], it has been suggested that mitochondria may have both pro-ferroptosis and anti-ferroptosis functions in a context-dependent manner [84]. A better understanding of mitochondrial regulation of ferroptosis will benefit both basic research in cell biology and clinical treatment of ferroptosis-related diseases.

#### 3.4.1. The Role of Mitochondria in Promoting Ferroptosis via ATP Generation and Inactivation of AMPK

The major function of mitochondria is to generate ATP via electron transport through the ETC complexes. Recent studies have shown that under an energy-depleted condition (such as glucose starvation), the energy sensor AMP-activated protein kinase (AMPK) is activated and consequently suppresses ferroptosis [82,83]. The underlying mechanism has been attributed to AMPK phosphorylation and inactivation of acetyl-CoA carboxylase (ACC). ACC is a PUFA synthesis rate limiting enzyme that mediates conversion of acetyl-CoA to malonyl-CoA, a precursor for PUFA synthesis. Therefore, the activation of AMPK suppresses lipid peroxidation and ferroptosis [82,83]. Consistent with these findings, various ETC complex inhibitors or mitochondrial uncoupling agents significantly suppress ferroptosis [81]. These results suggest that mitochondria may play an essential role in promoting ferroptosis, possibly through generating ATP and the subsequent inactivation of AMPK [81,82,83] (Figure 2; pro-ferroptosis). Additionally, as aforementioned, mitochondria are a major source of cellular ROS. The electron leakage from ETC complexes I and III produces superoxide O_2_^•−^. O_2_^•−^ is converted to H_2_O_2_ (by superoxide dismutase, SOD) and subsequently to hydroxyl radicals (^•^OH) (via the Fenton reaction). Hydroxyl radicals (^•^OH) are required for the initiation of lipid peroxidation [5,85]. Therefore, mitochondria likely contribute to the induction of ferroptosis by promoting lipid peroxidation in part via ROS production. The TCA cycle and glutaminolysis in mitochondria may promote ETC activities and further promote ferroptosis.

#### 3.4.2. The Role of Mitochondria in Suppressing Ferroptosis via mGPX4 and DHODH

While contradictory data have been obtained from previous studies on mitochondrial defense systems against ferroptosis, a recent study by Mao has provided solid evidence demonstrating that dihydroorotate dehydrogenase (DHODH)-CoQ10 together with mGPX4 serve as mitochondrial localized defense systems that suppress mitochondrial lipid peroxidation and consequently protect against ferroptosis. DHODH is a mitochondrial enzyme that catalyzes pyrimidine biosynthesis, where it couples the oxidation of dihydroorotate (DHO) to orotate (a precursor for pyrimidine synthesis) to the reduction of CoQ10 to CoQ10H2 [86,87]. CoQ10H2 subsequently acts as an RTA to reduce lipid ROS in the mitochondrial inner membrane. It has been shown that DHODH and mGPX4 form two major defense arms to suppress lipid peroxidation in the mitochondrial membrane and that it is necessary to disable both arms to sufficiently promote mitochondrial lipid peroxidation and to induce ferroptosis (Figure 2; pro-ferroptosis). Supporting this notion, Mao et al. showed that RSL3 treatment (GPX4 inhibition) in DHODH KO cells induced mitochondrial lipid peroxidation and ferroptosis, which were rescued only by overexpression of mGPX4, but not cGPX4 or FSP1 [70]. On the contrary, a high-dose RSL3-induced ferroptosis in wild-type cells was mainly mediated by cGPX4 inhibition and plasma membrane lipid peroxidation. Therefore, there are two separate defense systems in the cytosol (cGPX4/FSP1) and mitochondria (mGPX4/DHODH) that detoxify lipid peroxidation and defend against ferroptosis in cells [70,84].

### 3.5. Ferroptosis Inducers and Inhibitors

#### 3.5.1. Classes of Ferroptosis Inducers

Ferroptosis inducers (FINs) are compounds or treatments that can induce ferroptosis. Based on their targets and mechanisms of action, FINs have been categorized into at least four classes as follows [88]:

- Class I FINs (e.g., erastin, sulfasalazine, and sorafenib) are blockers of System x_c_^−^ (i.e., SLC7A11). They prevent cystine import and deplete intracellular GHS levels, thereby reducing GPX4 activity [7]. FINs may be useful as a strategy for the treatment of certain cancers that are susceptible to ferroptosis.
- Class II FINs (e.g., RSL3) are direct inhibitors of GPX4, resulting in the loss of its enzymatic activity and thereby reducing lipid peroxide content [7,16].
- Class III FINs (e.g., FIN56) act by depleting GPX4 protein and CoQ10 simultaneously, thereby causing accumulation of lipid peroxide.
- Class IV FIN (FINO2) acts by oxidizing iron and indirectly inactivating GPX4, thereby driving lipid peroxidation [89]

It should be noted that iron treatment (e.g., ferric ammonium citrate) per se is sufficient to cause ferroptosis in cardiomyocytes [34,52,53] and can be used as an in vitro model of iron overload-induced ferroptosis.

#### 3.5.2. Ferroptosis Inhibitors

Inhibiting ferroptosis may have clinical potential to serve as a treatment approach for some types of degenerative diseases (e.g., Alzheimer’s and Parkinson’s disease) as well as cardiovascular disease as discussed in Section 4. Most often-used ferroptosis inhibitors are iron chelators and lipophilic RTAs. Iron chelators such as deferoxamine (DFO), dexrazoxane (DXZ), and ciclopirox reduce labile iron levels and suppress the Fenton reaction and LOX (an iron-dependent enzyme) activity, thereby preventing the initiation and propagation of lipid peroxidation. On the other hand, lipophilic RTAs can trap chain-carrying radicals and thereby block the propagation of radical chain reactions during lipid peroxidation [90]. Ferrostatin-1 (Fer-1) and liproxstatin-1 are two widely used lipophilic RTAs that inhibit ferroptosis [91], which are considered as effective and reliable tools to examine whether a given cell death is indeed ferroptosis [7]. Furthermore, CoQ10H2, which can be produced by FSP1 or DHODH from CoQ10, is a naturally occurring common RTA that is capable of inhibiting ferroptosis. Interestingly, a very recent study by Mishima et al. has reported that vitamin K (VK) prevents ferroptosis via a FSP1-dependent reduction pathway. VK shares structural similarities with CoQ10. The authors revealed that FSP1 reduces VK to VKH2, which acts as an antioxidant, eliminating radicals and preventing lipid peroxidation [92].

## 4. Implications of Ferroptosis in Cardiovascular Disease

**Figure 4:**
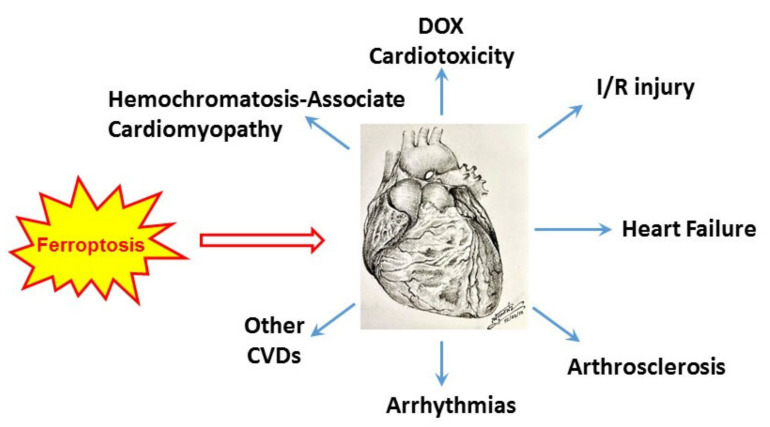
Possible implication of ferroptosis in cardiovascular disease. DOX: doxorubicin; I/R: ischemia-reperfusion; CVD: cardiovascular disease. The sketch of heart was a generous gift from Dr. Natthaphat Siri-Angkul.

Recent studies have linked ferroptosis to various pathological conditions. For example, impaired ferroptosis has been associated with tumor development and progression [93,94,95], while excessive ferroptosis has been recognized as the cause of neurodegenerative diseases (e.g., Alzheimer’s and Parkinson’s diseases), ischemia/reperfusion (I/R)-induced organ injury, as well as various other forms of disease (e.g., infectious disease) [96,97,98,99,100]. Emerging evidence has revealed that ferroptosis plays an essential role in the development of a diverse range of cardiovascular diseases [37,38,101,102]. Conversely, ferroptosis inhibitors, iron chelators, antioxidants and genetic manipulations may alleviate myocardial injury by blocking ferroptosis pathways. In this section, we summarize recent progress in the implication of ferroptosis in diverse cardiovascular diseases (CVDs) (Figure 4).

### 4.1. Hemochromatosis-Associated Cardiomyopathy

While iron is required for normal physiological processes, it can become toxic when iron overload occurs, such as in patients with hereditary forms of hemochromatosis or as a result of multiple blood transfusions as seen with sickle cell disease or in patients with β-thalassemia [12]. The heart is one of the major target organs for iron deposition which can be manifested as iron overload cardiomyopathy (IOC) [103,104]. IOC is the leading cause of morbidity and mortality in patients with primary hemochromatosis and secondary iron overload. These patients exhibit cardiomyopathy as well as life-threatening cardiac arrhythmias. Although IOC remains a global tragedy and significant clinical challenge in the U.S. and internationally, the underlying mechanism(s) involved in IOC iron-associated cardiotoxicity have not been well defined [12,104,105].

Ferroptosis has recently been implicated in iron overload-induced cardiac cell death and cardiovascular disease [34,106,107,108]. Baba et al. have demonstrated that ferric iron-citrate, erastin, and RSL3 readily induce ferroptosis in cultured adult mouse cardiomyocytes, which can be reversed by the ferroptosis inhibitor ferrostatin-1 [34]. As previously discussed (Section 3.2), iron catalyzes the Fenton reaction, resulting in the formation of soluble peroxides or lipid peroxides that then generate toxic hydroxyl or lipid alkoxyl radicals, which can then propagate lipid peroxidation. Baba et al. further demonstrated that the mechanistic target of rapamycin (mTOR) plays an important role in protecting cardiomyocytes against excess iron and ferroptosis, probably by regulating ROS production [34].

It is well known that mitochondria play an important role in the process of iron metabolism. Iron overload may occur at the cellular as well as mitochondrial level. It should be noted that mitochondrial iron overload may play an important role in causing mitochondrial dysfunction and even ferroptosis [12,53,109,110]. We have found that iron overload promotes ROS production, depolarizes mitochondrial membrane potential (Δ*Ψ*_m_), and disrupts cytosolic Ca dynamics. Cytosolic Ca dysregulation is a recognized hallmark of cardiac dysfunction and heart failure [111]. The involvement of the mitochondrial permeability transition pore (mPTP) has been suggested as contributing to cytosolic Ca dysregulation [112]. Kumfu et al. have shown that mCU is involved in mitochondrial dysfunction under iron-loaded conditions [109]. We [53] have also demonstrated that iron overload-induced ferroptosis is dependent on the mitochondrial Ca uniporter (mCU) using a conditional mCU KO mouse model created by Kwong et al. [113]. Supporting the importance of mitochondrial labile iron in the induction of ferroptosis, it has also been reported that overexpression of the iron-storage protein ferritin in mitochondria suppresses erastin-induced ferroptosis [114]. However, detailed mechanisms for mitochondrial iron metabolism in the regulation of mitochondrial (dys)function and ferroptosis, such as the role of mCU, crosstalk between Ca and iron, compartmentalization of lipid peroxides, etc., are still not well understood. Nevertheless, preventing cellular and mitochondrial iron overload by either reducing iron uptake or increasing iron storage, e.g., via transferrin, may inhibit the occurrence of ferroptosis. Iron chelators have also been widely used to treat IOC [12].

### 4.2. Cardio-Oncology: Doxorubicin-Induced Cardiomyopathy

Doxorubicin (DOX) is a widely used anthracycline anticancer agent. However, its clinical efficacy is limited by cardiotoxicity referred to as doxorubicin-induced cardiomyopathy (DIC). The detailed mechanisms for DIC and its therapeutic strategy have yet to be fully elucidated. Previous studies have linked Ca handling abnormalities, mitochondrial iron accumulation, and mitochondrial dysfunction to DIC [115,116,117,118]. It has also been suggested that several RCDs such as apoptosis, necroptosis, and autophagy are involved in DOX-induced cardiomyocyte death [115,116,119,120,121]. Several recent studies have recently demonstrated that ferroptosis is likely to be the major form of RCD in DOX-induced cardiomyocyte death. Therefore, therapeutics targeting ferroptosis might be a novel preventive strategy for DIC [35,80,122,123,124]. It has been demonstrated that heme oxygenase 1 (Hmox1)-dependent heme degradation and free iron overload promote ferroptosis and DIC [35]. Furthermore, doxorubicin has been shown to suppress mGPX4 expression thereby inducing excessive lipid peroxidation in mitochondria and might consequently lead to mitochondria-dependent ferroptosis. Furthermore, the overexpression of mGPX4 or iron chelation targeting mitochondrial iron content significantly rescued doxorubicin-induced ferroptosis [80]. Whether doxorubicin also suppresses the expression or activity of DHODH, another arm of the mitochondrial lipid peroxidation defense system, remains to be studied.

### 4.3. Cardiac Ischemia/Reperfusion (I/R) Injury

It has been observed that myocardial injury may lead to the release and accumulation of local iron [125]. Previous studies have suggested that mitochondrial iron is involved in cardiac ischemic events [126]. Iron regulatory proteins such as transferrin receptor 1 [127] and ferroportin [128] have been shown to be associated with myocardial I/R injury. Furthermore, the overexpression of GPX4 in mitochondria suppresses I/R injury and reduces lipid peroxidation [129], suggesting a potential association to ferroptosis. A number of very recent studies have further revealed cardiac I/R injury may cause the disruption (expression and activation) of the GPX4 system as well as trigger the accumulation of lipid peroxidation (such as oxidized phosphatidylcholines), thereby inducing cardiomyocyte ferroptosis [35,39,130,131,132,133]. In addition, the level of NOX2 (thus generation of ROS) has been shown to also be increased during myocardial I/R injury [134]. Moreover, it has been demonstrated that the Nrf2 pathway contributes to ferroptosis in I/R injury [135]. Administration of Fer-1 reduced infarct size caused by I/R injury and showed a long-term improvement in cardiac function [136]. Therefore, ferroptosis is associated with cardiac I/R injury as a significant form of cell death in cardiomyocytes, although it should be noted that apoptosis and necroptosis have also been observed in I/R injury.

### 4.4. Heart Failure

Heart failure (HF) is a serious condition characterized by significantly reduced cardiac output and cardiomyocyte cell death [111]. Recent studies have linked ferroptosis to HF induced by genetic manipulation in addition to receiving a diet high in iron content and the presence of pressure overload. Fang et al. [36] has demonstrated that the iron storage protein ferritin H (Fth) plays a central role in mediating cardiac iron homeostasis and protecting against cardiac ferroptosis and subsequent cardiomyopathy (even HF) induced by a diet with a high iron content. Fth-deficient mice showed increased ROS production and developed HF at 6 months of age with a high-iron diet. HF could have been ameliorated by either Fer-1 or by overexpression of x_c_^−^ (SLC7A11), which increased GSH levels and suppressed ferroptosis. Furthermore, ferroptosis was found to play an important role in a rat HF model induced by transverse aortic constriction (TAC) [64]. Knocking-down Toll-like receptor 4 (TLR4) or NOX4 remarkably reduced the incidence of ferroptosis and improved heart function, suggesting that TLR4-NOX4 might be a potential therapeutic target for HF at least partly through inhibiting ferroptosis-mediated cell death. Moreover, in a TAC-induced rat HF model, Liu et al. [137] suggested that puerarin, an isoflavone, may protect against HF induced by pressure overload by decreasing lipid peroxidation and mitigating ferroptosis.

### 4.5. Atherosclerosis

Atherosclerosis is a chronic inflammatory disease of arteries characterized by disorders of lipid metabolism. It has been previously realized that GPX4 and lipid peroxidation play significant roles in atherosclerosis. For example, Guo et al. [138] demonstrated that overexpression of GPX4 decreased lipid peroxidation and alleviated atherosclerotic lesions in the aorta of ApoE-deficient mice. Considering that reduced GPX4 expression and/or activation and increased lipid peroxidation are the main features of ferroptosis, recent studies [139] have explored the potential association between ferroptosis and atherosclerosis.

Bai et al. found that Fer-1 partially reduced iron accumulation, lipid peroxidation, and reversed the expressions of SLC7A11 and GPX4 in aortic endothelial cells in high fat diet-fed ApoE(−/−) mice [139]. Fer-1 reduced high fat diet-induced atherosclerotic lesions in ApoE-deficient mice by reducing lipid peroxidation. Furthermore, oxidized-low density lipoprotein (ox-LDL)-induced cell death was also examined in mouse aortic endothelial cells (in vitro). More importantly, Fer-1 reduced cell death in ox-LDL-treated cells, suggesting the involvement of ferroptosis during the initiation and development of atherosclerosis. In addition, ferroptosis has also been linked to atherosclerosis in human coronary artery specimens by analyzing the correlation to ferroptosis markers such as PTGS2 (upregulated), ACSL4 (upregulated), and GPX4 (downregulated) [140].

### 4.6. COVID-19 Associated Arrhythmias

Both excessive ROS generation and iron overload have a close association with arrhythmogenesis [12,73,112,141]. However, the implication of ferroptosis in arrhythmogenesis has not been investigated. It has been suggested that cardiac arrhythmias are one of the clinical features of coronavirus disease 2019 (COVID-19). An earlier study by Jacobs et al. observed lipid peroxidation in the myocardium and suggested that ferroptosis may be a detrimental factor in cardiac damage resulting from COVID-19 [142]. A recent study [143] investigated the potential role of ferroptosis-induced sinus node damage in causing cardiac arrhythmias. The study used human embryonic stem cell (hESC)-derived sinoatrial node (SAN)-like pacemaker cells and showed that severe acute respiratory syndrome coronavirus 2 (SARS-CoV-2) infection induced ferroptosis and caused the dysfunction of human SAN-like pacemaker cells. This result suggests that ferroptosis is a potential mechanism for inducing arrhythmias in patients with COVID-19. Iron chelators and/or ferroptosis inhibitors might be able to block SARS-CoV-2 infection and be used to treat cardiac arrhythmias as well as other CoV-2–associated organ failures.

## 5. Conclusions

Having common features that related to ferroptosis, such as iron accumulation, increased production of ROS, and lipid peroxidation, more and more CVDs have been associated with ferroptosis by recent intensive studies including ours [6,39,52,53]. In addition to those listed in Section 4, these CVDs also include Friedreich’s ataxia associated cardiomyopathy [144], sickle cell disease associated cardiomyopathy [145], and myocarditis (such as SARS-CoV-2 infection) [142]. Potential involvement of ferroptosis in other CVDs such as lysosomal storage disease related cardiomyopathy [146] remains to be elucidated.

Notably, increasing evidence has suggested that there may exist crosstalk between ferroptosis and other types of cell death such as autophagy and apoptosis [147,148]. However, there are no specific in vivo biomarkers for ferroptosis [2]. Discovery of specific biomarkers of ferroptosis may be useful for detecting ferroptosis at the tissue as well as systemic level and potentially help in diagnosis, treatment, and prognosis for CVDs in the future.

In conclusion, ferroptosis plays a critical role in the pathogenesis of various CVDs and the suppression of ferroptosis is expected to become a potential therapeutic option for multiple CVDs. The candidate agents for ferroptosis suppressors include iron chelators (e.g., DFO and DXZ) and selective ferroptosis inhibitors (e.g., the lipophilic RTAs Fer-1 and liproxstatin-1). In addition, pharmacological drugs or genetic manipulations that down-regulate TfR or up-regulate ferroportin, SLC7A11, or GPX4 expression/activity may provide protection against ferroptosis and multiple forms of CVD.

## Data Availability

Not applicable.
